# The good, the bad, and the red: implicit color-valence associations across cultures

**DOI:** 10.1007/s00426-022-01697-5

**Published:** 2022-07-15

**Authors:** Claudia Kawai, Yang Zhang, Gáspár Lukács, Wenyi Chu, Chaoyi Zheng, Cijun Gao, Davood Gozli, Yonghui Wang, Ulrich Ansorge

**Affiliations:** 1 Department of Cognition, Emotion, and Methods in Psychology, Faculty of Psychology, University of Vienna, Liebiggasse 5, 1010 Vienna, Austria; 2 School of Psychology, Shaanxi Normal University, Xi’an, China; 3 Nexus International School, Putrajaya, Malaysia; 4 Department of Psychology, Central China Normal University, Wuhan, China; 5 Department of Psychology, University of Macau, Zhuhai, China; 6 Vienna Cognitive Science Hub, University of Vienna, Vienna, Austria; 7 Research Platform Mediatised Lifeworlds, University of Vienna, Vienna, Austria

## Abstract

Cultural differences—as well as similarities—have been found in explicit color-emotion associations between Chinese and Western populations. However, implicit associations in a cross-cultural context remain an understudied topic, despite their sensitivity to more implicit knowledge. Moreover, they can be used to study color systems—that is, emotional associations with one color in the context of an opposed one. Therefore, we tested the influence of two different color oppositions on affective stimulus categorization: red versus green and red versus white, in two experiments. In Experiment 1, stimuli comprised positive and negative words, and participants from the West (Austria/Germany), and the East (Mainland China, Macau) were tested in their native languages. The Western group showed a significantly stronger color-valence interaction effect than the Mainland Chinese (but not the Macanese) group for red-green but not for red–white opposition. To explore color-valence interaction effects independently of word stimulus differences between participant groups, we used affective silhouettes instead of words in Experiment 2. Again, the Western group showed a significantly stronger color-valence interaction than the Chinese group in red-green opposition, while effects in red–white opposition did not differ between cultural groups. Our findings complement those from explicit association research in an unexpected manner, where explicit measures showed similarities between cultures (associations for red and green), our results revealed differences and where explicit measures showed differences (associations with white), our results showed similarities, underlining the value of applying comprehensive measures in cross-cultural research on cross-modal associations.

## Introduction

Humans form associations between colors and affective valence, such as between black and negative valence versus white and positive valence (Lakens et al., 2013). These associations are important for many different applications. For instance, coloration of relevant signals for machine users (e.g., coloring of traffic lights, on–off buttons, etc.) could be designed in a more or less intuitive way (Garrido et al., 2019; Hochman et al., 2018). Critically, such signals are typically based on color systems, in which at least two different colors take on different, oftentimes even opposing, functions or meanings. For example, in some countries, white characters on blue or green backgrounds are used for traffic signs for guidance, while red circles are used for regulation signs. A very conventional system uses green or white traffic lights for signaling go and red for signaling stopping actions. In addition, buttons—physically on machines or virtually on the Internet and mobile applications—systematically use the opposing “call-to-action” colors red for *cancel*, *error*, and *decrease*, and green for *ok*, *success*, and *add*. It is also common to mark gaming tokens or playing cards by different colors (e.g., black and white chess pieces, or red vs. black for uneven vs. even numbers in roulette).

Importantly, for the cross-cultural success of such applications, underlying associations between color and valence could apply universally or they could differ between cultures. Studies that used explicit ratings of color-emotion or color-valence associations found more cross-cultural similarities than differences (e.g., Adams & Osgood, 1973; Barchard et al., 2017; Hupka et al., 1997; Jonauskaite et al., 2020a; Volkova et al., 2012; Wang et al., 2014). Such universal color-valence or color-emotion associations could reflect communalities between globally shared knowledge (i.e., a global or globalized culture) but they could also be of an evolutionary origin (for the logic, see Darwin, 1872; Ekman & Friesen, 1975).

Critically, what is currently lacking are more implicit measures of the cultural universality of these color-valence associations to complement the findings obtained with explicit measures. To start with the importance of implicit measures, although explicit ratings and judgments carry a high face validity and allow for an economical and encompassing measure of the different emotions associated with each color, explicit ratings also have a number of drawbacks (see, e.g., Specker & Leder, 2018). Most notably, they depend on the participants’ awareness of the associations, meaning also that participants need to remember these associations explicitly to report them in the first place (Squire, 1986). Explicit ratings are, thus, not always suited to tap into the more implicit forms of memory, including associations built on conditioning, for example (cf. Squire, 1986; Squire et al., 1993).

### Color associations in China and the West

Generally, when it comes to color-emotion associations, explicit and implicit measures show similar results. For example, red has shown its character as a highly affective but emotionally ambiguous color in explicit naming as well as implicit association studies: prominently named associations like ‘anger’ as well as ‘love/passion’ (Jonauskaite et al., 2020a; Kaya & Epps, 2004) were corroborated with implicit measures (see Elliot & Niesta, 2008; Elliot et al., 2010; Fetterman et al., 2012; but see Lehmann et al., 2018; Peperkoorn et al., 2016; see also implicit associations for red with danger, Pravossoudovitch et al., 2014; failure, Moller et al., 2009, and high social status, Wu et al., 2018).

However, the situation is different with respect to cultural differences. As of today, we can say little about the cross-cultural differences versus similarities between implicit measures of color-valence associations; while we have a solid body of research on explicit color associations from different cultures around the globe, there has been little inquiry into cross-cultural difference and similarities in *implicit* measures. Among the few contributions in this area is, apart from the paper by Wu et al., (2018, see above), the comparison of implicit associations for the colors red and green between Mainland (ML) China and Hong Kong by Jiang et al. (2014). This article showed opposite spatial associations between the two culture groups, demonstrating a red-up/green-down congruence for Mainland Chinese and a green-up/red-down association for Hong Kong Chinese participants. In addition, the results suggested a generally greater positive perception of the color red in ML China as compared to a more ‘Westernized’ culture in Hong Kong (This fact additionally draws attention to the significance of specifying a ‘Chinese’ sample in more geographic detail when presenting research results or making predictions. We will get back to this point further below).

For most of the research, however, cross-cultural comparisons of implicit measures of color-valence association are lacking. For instance, many studies using explicit measures showed cultural similarities for associations to green color, which are generally largely positive around the globe (e.g., Adams & Osgood, 1973; Jonauskaite et al., 2020a). Interestingly, for the color white, explicit measures showed a cultural difference: In Western cultures, white carries mostly positive connotations, but in China, white carries also negative connotations of ‘sadness’ (e.g., Jonauskaite et al., 2019, 2020a; see also Wang, 2013). This gives rise to the assumption that white might be perceived as a more ambiguous color in Eastern cultures (but see Saito, 1996; Zhang et al., 2019).

However, while evidence from explicit associations is, for the most part, well corroborated by *implicit* measures, this is typically shown for Western samples only (e.g., Meier et al., 2004; Schietecat et al., 2018a, 2018b; here see also Lakens et al., 2012)—a cross-cultural comparison of these implicitly measured associations is lacking (but see Specker et al., 2018).

### The significance of color systems

In their implicit measures, Schietecat et al., (2018a, 2018b) explored the influence of polar color oppositions with respect to different emotional dimensions (aka the *dimension-specificity hypothesis*). To start with, emotions are defined by a number of dimensions, such as valence or arousal (cf. Russell, 1980; Wundt, 1874). In a series of implicit association tests (IAT), Schietecat et al., (2018a, 2018b) showed that color opposition context influences (1) the strength of the valence association of the color red (i.e., a stronger congruence effect in a red-green opposition than in a red-blue opposition), and (2) the predominantly associated emotional dimension (i.e., associations between ‘negative valence’ and red in red-green opposition versus associations between ‘activation’ and red in red-blue opposition).

These influences of color opposition, or *color systems* as a contextual factor for color-valence associations are highly relevant in applied ecological settings. Kawai (2021) defines color systems in the following way:

“With the term color systems we refer to the use of more than one color, repeatedly appearing within a certain context. Typically arising from the specific use of these colors in relation to one another (e.g., communicative functions), specific message-signaling calcifies by association. Encountering a color within its color system (e.g., red within a red-green color system) may highlight the respective associations (e.g., red as negative signal to green). Importantly, these color-system related associations may not be salient in other contexts (e.g., isolation) or color systems (e.g., red-white).” (p. 11)

It is immediately apparent that color opposition is a practical concept directly used in designing human–machine interfaces and communication systems (e.g., in traffic lights). Here, we investigated two often used and, thus, very relevant color opposition systems: red–green and red–white.

Red–green color systems are very prevalent in Western cultural environments (see previous sections) and implicit associations are in accordance with the communicative function the colors serve to express within this system (e.g., green-positive/red-negative, see Kawai et al., 2020; green-safety/red-danger, Pravossoudovitch et al., 2014). However, findings from Jiang et al. (2014), for instance, call into question whether associations for red and green will go in the same direction in Eastern and Western culture. Testing directly for cross-cultural differences in implicit color-valence associations (instead of cross-modal color-space relations) seems necessary in the face of these prior results.

As explained by Schietecat et al., (2018a, 2018b), specific colors take on different roles or meanings in different contexts (cf. Elliot & Maier, 2012), and the specific color used as an alternative provides a particular context that could be decisive for which color-valence association dominates. To study the role of such color systems in a cross-culturally varying context, we will contrast the red–green opposition to a red–white opposition. As mentioned above, we selected white for its already demonstrated cultural differences (sadness associations, e.g., Jonauskaite et al., 2019). Additionally, there are arguments for a prominent red–white color system in traditional Chinese culture (see, e.g., the art of calligraphy or seal-cutting). Specifically, He (2011) argued that in “Chinese culture, white is contrary to red” (p. 161). This color symbolism is reflected in language and culture. In the Beijing opera, for example, the hero wears a red face mask and the adversary a white one (China National Tourist Office, 2020). The symbolism of red as the color of luck and prosperity and white as a color associated with mourning is further recognizable in traditions such as posting red colored couplets on windows and doors during the Spring Festival, while posting white colored ones when a death occurred in the family (Ibekwe, 2021). Another illustrative example is the custom of wearing red clothes for weddings and white clothes for funerals. In fact, in Simplified Chinese, the term for *wedding*, 红事 (hóng shì), is composed of the constituents *red* (红, hóng) and *matter* (事, shì). Opposed to that stands the word for *funeral*, 白事 (bái shì), which is a compound of *white* (白, bái) and *matter* (事, shì).This is not the case in Western languages such as English, German, French, or Spanish.

### Predictions and the congruence effect measure

For each of the two color systems (red–green; red–white), we used an implicit measure and tested processing of two valence categories (positive vs. negative). This was done in a valence categorization task of target objects of different colors (e.g., positive and negative words presented in red and green in Experiment 1, positive and negative images presented in red and green in Experiment 2). Predictions for the resulting 2 (colors) × 2 (valences) factorial design of our study were based on congruence relations or assumed associations between colors and valence in ‘Western’ populations: Accordingly, green-positive, white-positive, and red-negative are congruent pairs—expected to facilitate processing, while green-negative, white-negative, and red-positive are incongruent (or less congruent) pairs—expected to delay processing. Naturally, we expected categorization of targets by their valence to be faster and more accurate in congruent pairs than incongruent pairs (*congruence effect*, CE). In our analyses, we compared CEs between cultures to follow up on a significant Color × Valence × Country interaction.^1^ In mathematical terms, the CE is the average difference between the mean performance in the incongruent condition minus the mean performance in the congruent condition. In the present context, a CE difference between cultures tells us if and in how far a culture’s congruence effect and, hence, an underlying color-affect association, deviates from the ‘Western’ definition of this congruence.

*Concerning our predictions* If no cultural differences are revealed using implicit measures (here CEs), this would suggest that the cultural differences found in explicit measures reflect culture-specific associations, whereas implicit measures tap more into phylogenetically shared roots of color-valence associations or into that part of the experience-based color-valence associations that is shared across cultures, for instance, because a corresponding color system corresponds to an internationally applied convention. In this case, the CE will not differ between cultures in either color opposition condition.

If results from implicit measures mirror those from explicit measures, then we would expect culture-driven differences in CE to be stronger in the red–white rather than the red–green color opposition condition, due to the selectively stronger white-sadness connotations for Chinese over Western participants (see He, 2011; Jonauskaite et al., 2020a). The most extreme difference would occur with inversion of congruence (what is considered *incongruent* in the Western group is *congruent* in China, e.g., white-negative/red-positive in China), reflected in a negative CE in China (since congruence would be defined as white-positive/red-negative).

However, should this not be the case and results with our implicit measure diverge from the predictions we derived from explicit measures, then we successfully showed that implicit measures are a useful tool to uncover differences between the cultures that more explicit measures might be insensitive to.

### Current study

For operationalization of our implicit measures of color-valence associations, we used varying font colors (cf. Jonauskaite et al., 2020b) rather than color words (cf. Jonauskaite et al., 2020a), as the usage of physical color allows more control over what participants actually see and incorporate into their judgments.^2^

In the first experiment of the current study, we tested color-valence associations implicitly in a participants from a Western population (Austria) and compared it to an Eastern culture from Mainland China as well as to a sample “in-between” Eastern and Western cultures from Macau—now a part of China, but a former Portuguese colony (until 1999). We used positive and negative words in either red or green color or in either red or white color, and we asked our participants to categorize the words by their valence (as positive or negative).

In Experiment 2, we used pictures rather than words. Like words, pictures can reliably signify emotional content. Complex photorealistic pictures as well as black-and-white outline drawings or silhouettes have been shown to elicit emotional responses (e.g., Bradley & Lang, 2007; Giner-Sorolla et al., 1999; Schimmack, 2005). Since it is impossible to control for equivalence in all characteristics of translated words between different languages (e.g., their lengths, their transparencies, their orthographic neighbors, etc., as used in Experiment 1), we used pictorial stimuli in Experiment 2 that were genuinely identical and thus comparable. We used simple silhouettes rather than realistic photographs, as it was easier to manipulate the colors of the silhouettes without corrupting their meaning altogether than it would have been the case with photos. In addition, silhouettes are not that rich in visual detail referring to flora, fauna, objects, buildings, landscape, weather conditions, traffic, clothing, etc., and, thus, they allow more control with respect to specific cultural content than photographs. Two groups of participants were tested online, a Mainland Chinese sample and a predominantly German sample.

### Sample size determination

For Experiment 1, the sample size was based on the considerations reported in Kawai et al. (2020), where sample size was determined from the effect size observed in a pilot study by Lohmann and Jorschick (2015) resulting in a minimum sample of 20 participants for a within-participants interaction. The actual sample collected in Kawai et al. (2020) for the red–green mixed block comprised 45. This number constituted the minimum number of participants per cell (Country × First Color Opposition Block Condition = 3 × 2 = 6), resulting in at least 45 × 6 = 270 participants. Since we lacked effect size data from comparable studies for Experiment 2, but anticipated the possibility of weaker interactions in pictorial as opposed to linguistic material, we increased the minimum number of participants per cell to 60 (Country × First Color Opposition Block Condition = 2 × 2 = 4), resulting in a minimum of 60 × 4 = 240 participants.

## Experiment 1

### Methods

#### Participants

Data from 281 participants were collected; 104 in Austria (University of Vienna), 90 in Macau (University of Macau), and 87 in Mainland China (Shaanxi Normal University, Xi’an, China). Participants were randomly assigned to one of the conditions that resulted from permuting block order and key location.

The data from Austria was collected in two sets, owing to the fact that they stemmed from different studies. The Austrian red–green data was taken from the “mixed blocks” of Kawai et al. (2020), in which participants saw a monochromatic red, a monochromatic green, and a mixed red–green color block. The Austrian red–white data were taken from a preregistered study (https://osf.io/dfs9e/), in which participants saw a mixed red–white and a mixed red–black color block. Here, we only included data from the red–green mixed block and the red–white mixed block from those participants that saw this particular mixed block *first* in their experimental session (not as a second or third experimental block). This was done to forego any carry-over effects of other color opposition blocks. We do not consider color blocks that are not of interest for the current investigation (i.e., red and green monochromatic blocks, red–black opposition blocks). The red–green and red–white data in Mainland China and Macau were collected within the same study, so each participant in these groups saw both color opposition blocks. For these two groups, we followed the same procedure as for the Austrian group, namely considering only the first experimental block from each participant to avoid carry-over effects (for more information, see “Design” Section).

We excluded participants with a reported country of origin other than Germany or Austria for the German-speaking group (*n* = 10), Mainland China for the Chinese group (*n* = 1), and Macau for the Macanese^3^ group (*n* = 47). We also excluded all participants who did not reach the full score in the color-deficiency test or were self-reportedly color-blind (*n* = 4) and those who classified less than 40 stimuli per valence category in accordance with our valence assignment (*n* = 2, see *Procedure*). One participant had an accuracy rate lower than 75% (74.7%). From the 281 remaining participants, data from 65 people was excluded from analysis, leaving a total of 216 participants: group Austria with 91 subjects (*M*_age_ = 20.9 ± 2.8; 15 male), group Macau with 40 subjects (*M*_age_ = 19.6 ± 1.8; 15 male), and group China with 85 subjects (*M*_age_ = 18.9 ± 1.8; 15 male). Note that we will refer to the sample recruited in Austria as the "Austrian Group" and use the label "Austria" in the plots of Experiment 1 for simplicity's sake. The sample consisted of 58 Austrian and 33 German nationals.

#### Design

We investigated implicit color-valence associations in two different color opposition conditions: a red–green color-opposition block, and a red–white color-opposition block. Consequently, our sample was split into two block-order groups: one group starting with the red–green opposition block, the other group starting with the red–white opposition block. Results from these two participant groups were analyzed separately (reported in Sections “Red–green color opposition” and “Red–white color opposition”, respectively).

The way the data were collected (a subset from two different studies in Austria, see Kawai et al., 2020, and https://osf.io/dfs9e/; the full set for the groups from Macau and Xi’an) does not allow for a similar treatment of this factor. Color-Opposition Block with the two levels red–green and red–white was thus technically a between-subjects factor among the two Austrian study groups, while it constituted a within-subject factor for the two other country groups ML China and Macau. However, we did not run any comparative statistical analyses between the two color opposition blocks.

Thus, the factorial design of the statistical analyses comprised three factors: Country (Austria vs. China vs. Macau, between-participants) × Valence (positive vs. negative, within-participant) × Color (red vs. non-red, within-participants). As mentioned, the within-participant factor Color comprised the levels red and it’s opposing color, which, depending on the color opposition condition, was either green (for the group starting with red–green color-opposition block) or white (for the group starting with red–white color-opposition block). As dependent variables we collected response latencies (RTs) and accuracy (correct or incorrect valence classifications).

#### Materials

For the experimental groups in Austria, we used the same German words as stimuli as in Kawai et al. (2020). From the Berlin Affective Word List Reloaded (BAWL-R) database (Võ et al., 2009), 60 positive (mean emotion value ≥ 0.6) and 60 negative (mean emotion value ≤ – 0.6) German words were selected. The number of nouns, verbs and adjectives was balanced, and values for word arousal, imageability, letter count and word frequency (Cai & Brysbaert, 2010) were kept constant across the two valence categories. For the studies in China, the German word list was translated by a native Mandarin speaker to Mandarin (to be used in Mainland China, written in simplified Chinese characters) and Cantonese (used in Macau, written in traditional Chinese characters). This list was checked and verified by two native Cantonese and two native Mandarin speakers.

To ensure isoluminance for the stimuli presented in red and green, brightness values for red, green, and grey (background color) were measured with a spectrophotometer (X-Rite i1XTreme, Grand Rapids, MI, USA) for each of the five monitors used in the Austrian sample that completed the red–green mixed (i.e., opposition) condition. Color values were selected accordingly. For the samples from China and Macau, balanced RGB values for red (213, 0, 0) and green (0, 213, 0) were selected, and a medium gray (128, 128, 128). Since white has no chroma and is practically ultimate brightness of the monitor, equating values for hue and luminance in the red–white blocks was not feasible. We only had the chance to measure isoluminance of the displayed color values on the monitors in Austria but not at the test sites in China/Macau. However, color values were identical between the experimental sessions in China and Macau and lighting conditions were kept similar between all three countries. Additionally, the visual appearance of the colors on the monitors in the laboratories in China/Macau was judged by the first author of the present study to be sufficiently similar to that in the Austrian sample.

The complete stimulus list as well as a table with all colors and monitor resolutions that were used throughout the experiments are available in the online supplementary material.

In Austria, the size of the colored word stimuli was set to 50 pixels (angular size 1.45°), with a fixed viewing distance between eye and center display of 60 cm, through the utilization of chin rests. In the labs in China and Macau, chin rests were not available, so the participant’s chin was not fixated. However, the experimenter made sure to control that the setup had a viewing distance of approximately 50–60 cm.

#### Procedure

After signing the consent forms, participants were asked to provide demographic information (age, gender, country of origin). Instructions about the experimental procedure were then presented on screen. All text (instructions, stimuli, labels, etc.) was written in the participants’ native language. The study consisted of two tasks: an initial valence-rating task and a subsequent binary valence-classification task. The experimental session ended with a short test for color deficiency.

*Valence ratings* To ensure that every participant agreed with the valence category of a given stimulus, a rating task *(‘Please rate the valence of the word.’*) preceded the valence classification task. Participants judged each of 120 potential target words on a 10-point Likert scale (from ‘very negative’ to ‘very positive’) by moving the mouse cursor to the corresponding tick mark and confirming their selected valence value with a left-click. Words that received a rating below six were classified as ‘rated negative’ and appeared in a text box on the left side of the screen, below the negative scale label; words with a rating of six or higher were coded as ‘rated positive’ and appeared in a text box on the right side of the screen, below the positive scale label. Each word stayed on screen (centered above the rating scale) until the judgment was made. There were no time constraints for this task and participants were informed about this. After the participants rated all the words, the 50 most positively and the 50 most negatively rated words were selected for the upcoming valence-categorization task.^4^ The maximum of stimuli presented in the categorization task was 100, while there was no minimum of items specified in the experiment. However, per valence category (positive, negative), we set a lower boundary of at least 40 correctly rated words as participant exclusion criterion.

*Valence categorization* The binary categorization task started after participants had read the instructions on the monitor, which informed them that, per each trial, they would be presented with a single word (which they had previously seen in the valence rating task). Each target word was shown for a maximum of 2 s. For each word, participants judged the valence (*Is this word positive or negative?*) and indicated their choice by pressing either the ‘E’ (for positive valence) or the ‘I’ (for negative valence) key (key assignment was balanced across participants). We did not inform participants in advance that words would be presented in different colors. As mentioned above, the number of trials per participant could vary depending on the amount of their ‘valid’ valence ratings (those words rated in accordance with the specified valence categories, see “Material” Section). The maximum number of trials per participant per color block was 100 words × 2 presentation colors = 200 trials, the minimum (as specified by our exclusion criteria) was 80 × 2 = 160. The resulting average number of trials per participant was very close to the maximum, with 198.58 for the red–green blocks and 199.43 for the red–white blocks. The task started with a 10-trial practice. Participants from China and Macau completed two blocks—a red–green color block and a red–white color block (order of presentation was counterbalanced). As mentioned above, the data from the Austrian group stemmed from two distinct studies, one with red and green, the other with red and white stimuli (for details on the respective procedures see Kawai et al., 2020; as well as https://osf.io/dfs9e/). Stimuli were presented in randomized order, with the restrictions that no more than five words of the same valence and/or color were shown in a row. The duration varied among the participants, since the valence rating was self-paced, but generally, participants completed the entire experiment in less than 30 min, out of which the valence rating task took around 5–10 min and the valence categorization task around 15–20 min.

*Color vision test* After participants completed valence rating and valence categorization, we asked them to enter the numbers printed on three color plates, which were displayed on the computer screen (digitalized pictures of the Ishihara color plates, provided in the online supplementary material).^5^ Upon entering all three numbers, participants were debriefed in written form and the experiment ended.

#### Data analysis

For RT analyses, individual correct median RTs were averaged across participants, as the median is less sensitive to disproportionately slow responses than the mean. However, across participants, we calculated the mean of these median RTs. Trials with RTs below 150 ms and above 2 s were excluded from analysis (375 out of 67,776 trials, i.e., 0.6%; within the 67,401 timely responses, 4,206 were incorrect, i.e., 6.24%). To demonstrate the magnitude of the observed effects, partial eta-squared ($\eta_{{\text{p}}}^{2}$) values, 90% confidence intervals (CI), and generalized eta-squared ($\eta_{{\text{G}}}^{2}$) values are reported for *F*-tests (Steiger, 2004). We report Bayes factors (as BF_10_ when supporting difference, and BF_01_ when supporting equivalence) using the default *r*-scale of 0.707 (Morey & Rouder, 2018). In case of analyses of variance (ANOVAs), we report inclusion BFs based on matched models (Makowski et al., 2019; Mathôt, 2017). Where applicable, we report Welch-corrected *t*-tests (Delacre et al., 2017) with corresponding Cohen’s *d* values (Lakens, 2013). We used the conventional alpha level of 0.05 for all statistical significance tests. All analyses were conducted in R (R Core Team, 2019; via: Kelley, 2019; Lawrence, 2016; Lukács, 2020; Morey & Rouder, 2018).

### Results

#### Valence ratings

**Fig. 1:**
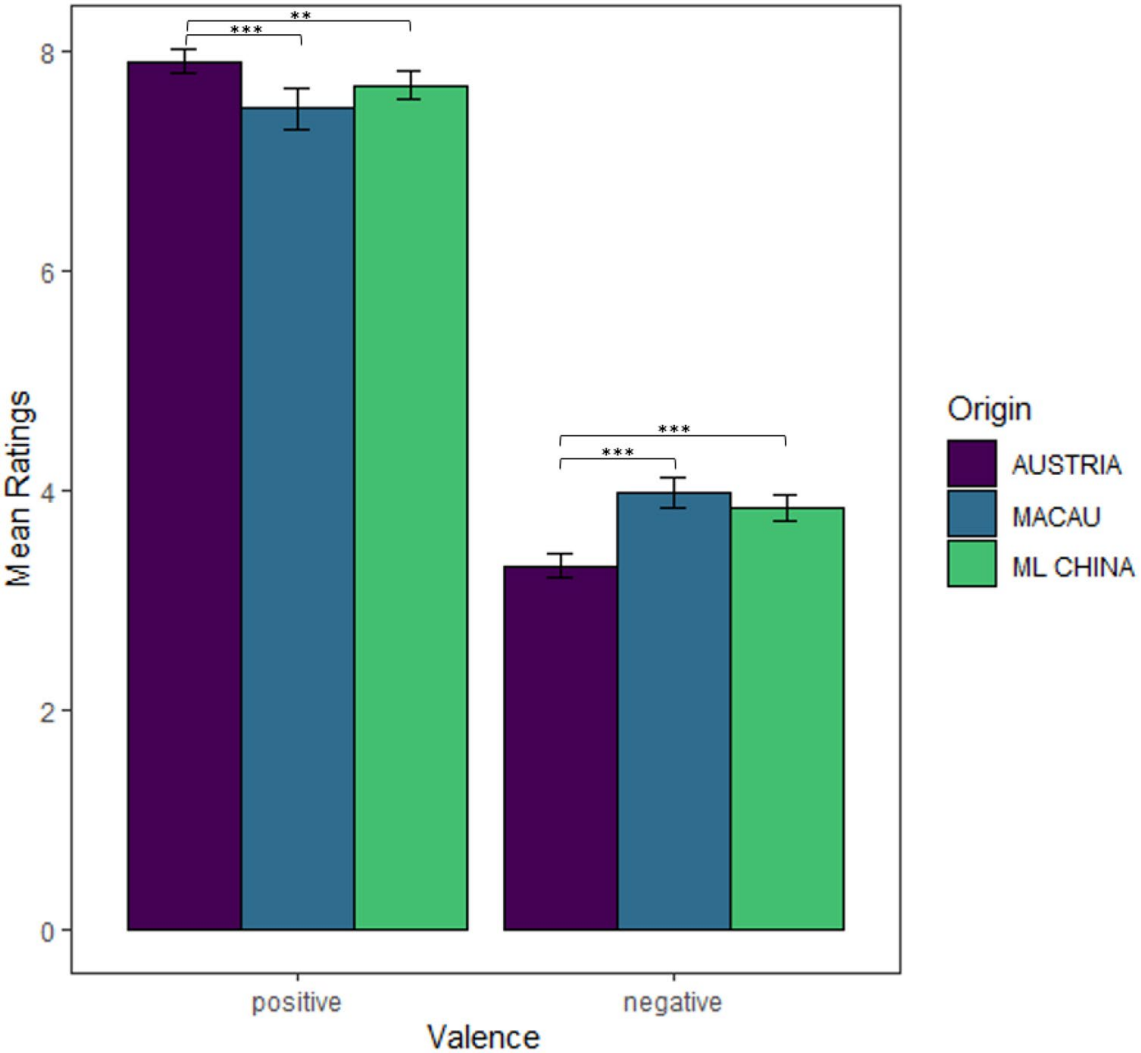
Mean ratings for positive and negative words in Experiment 1 by participants per country group. Error bars indicate 95% CIs of means. To illustrate significant group differences in the mean ratings, asterisks mark the *p* value of the *t*-tests reported above (*p < 0.017, **p < 0.01, ***p < 0.001)

We compared the correctly classified mean ratings per valence group (negative = 1–5, positive = 6–10) between the Austrian and the Chinese sample. The mean ratings were very similar in magnitude regardless of sample (Fig. 1). Nonetheless, we found some statistically significant differences (Bonferroni-corrected alpha level for a set of three *t*-tests is 0.017). For positive stimuli, there was evidence for a difference in the mean ratings (raw mean difference: 0.22, 95% CI [0.05, 0.39]), *t*(169.3) = 2.59, *p* = 0.010, *d*_between_ = 0.39, 95% CI [0.09, 0.69], BF_10_ = 3.60, with slightly lower (less positive) ratings in China (*M*_Rating_ ± SD = 7.68 ± 0.59) than Austria (7.91 ± 0.54). For the negative stimuli, ratings differed significantly (raw mean difference: – 0.54, 95% CI [– 0.70, – 0.37]), *t*(173.2) = – 6.55, *p* < 0.001, *d*_between_ = –0.99, 95% CI [– 1.30, – 0.67], BF_10_ = 1.42 × 10^7^, with lower (more negative) ratings by the Austrian (3.31 ± 0.54) than the Chinese sample (3.84 ± 0.54).

Interestingly, mean valence ratings between China and Macau did not differ significantly (positive valence: *M* ± *SD* = 7.47 ± 0.61 [Macau] vs. 7.68 ± 0.59 [China] and *t*[76.9] = –1.81, *p* = 0.074, *d*_between_ = – 0.35, 95% CI [–0.72, 0.03], BF_01_ = 1.11; negative valence: *M* ± *SD* = 3.98 ± 0.46 [Macau] vs. 3.84 ± 0.54 [China] and *t*(92.4) = 1.48, *p* = 0.143, *d*_between_ = 0.26, 95% CI [– 0.11, 0.64], BF_01_ = 2.09). Unsurprisingly, mean valence ratings between the groups Austria and Macau also showed significant differences (positive valence: raw mean difference of 0.43, 95% CI [0.21, 0.65], *t*(68.9) = 3.87, *p* < 0.001, *d*_between_ = 0.77, 95% CI [0.38, 1.14], BF_10_ = 260.88; negative valence: raw mean difference of – 0.67, 95% CI [– 0.85, – 0.49] and *t*(90.7) = – 7.37, *p* < 0.001, *d*_between_ = – 1.30, 95% CI [– 1.70, – 0.90], BF_10_ = 3.90 × 10^7^). Figure 1 illustrates the mean valence ratings for the Austrian, the Chinese, and the Macanese group.

#### Valence categorization

The correct median categorization times (RTs) were analyzed per color block. Note that in the Macau group, more than half of the participants were of non-Macanese origin (mainly students from China), which, after exclusion, left this participant group distinctly smaller than the two other groups. We will here report the analyses of the complete data set (three-leveled Country factor including Austria, China, Macau)^6^ because, in general, analyses showed very similar results (compared to a two-level factor comprising only Austria vs. China). The results of the two-country comparison (Austria vs. China) are available online in the supplementary material, together with the analyses of the error rates and two additional sets of preregistered supplementary statistical analyses (Block Order effects analysis, color-repetition trials analysis). In summary, these supplementary analyses largely confirm the effects found in the main analysis, which we will present here.

Below, we first report the results from the RT analyses of the red–green color block and then from the red–white color block. Aggregated means and *SD*s for the RTs in the different factor combinations can be found in Table 2.

##### Red–green color opposition

We analyzed RT data from the 110 participants who started the experiment with the red–green color-opposition block (44 from Austria, 43 from China, 23 from Macau) and ran a repeated measures ANOVA, with Country as a three-level between-participants factor (Austria vs. China vs. Macau), and Color (red vs. green) and Valence (positive vs. negative) as within-participant factors.

We found a significant main effect for Country, *F*(2, 107) = 24.01, *p* < 0.001, $\eta_{{\text{p}}}^{2}$ = 0.310, 90% CI [0.186, 0.406], $\eta_{{\text{G}}}^{2}$ = 0.287, BF_10_ = 2.39 × 10^6^, with faster responses in China (605.82 ± 71.74 ms) and Macau (637.06 ± 48.93 ms) than Austria (725.50 ± 81.09 ms). The main effect for Valence was significant, *F*(1, 107) = 69.37, *p* < 0.001, $\eta_{{\text{p}}}^{2}$ = 0.393, 90% CI [0.274, 0.489], $\eta_{{\text{G}}}^{2}$ = 0.029, BF_10_ = 9.98 × 10^11^, with faster responses to positive words (648.66 ± 88.62 ms) than negative words (672.15 ± 92.51 ms). Color did not influence RTs significantly, *F*(1, 107) = 3.46, *p* = 0.066, $\eta_{{\text{p}}}^{2}$ = 0.031, 90% CI [0, 0.102], $\eta_{{\text{G}}}^{2}$ = 0.001, BF_01_ = 4.47. Neither the Country × Color, nor the Country × Valence interactions reached significance (all *F*s < 2.00, all *p*s > 0.10). The Color × Valence interaction was significant, *F*(1, 107) = 105.43, *p* < 0.001, $\eta_{{\text{p}}}^{2}$ = 0.496, 90% CI [0.384, 0.580], $\eta_{{\text{G}}}^{2}$ = 0.033, BF_10_ = 14.82 × 10^17^. Importantly, this interaction was modulated by Country, expressed by a significant three-way interaction with *F*(2, 107) = 6.60, *p* = 0.002, $\eta_{{\text{p}}}^{2}$ = 0.110, 90% CI [0.026, 0.198], $\eta_{{\text{G}}}^{2}$ = 0.004, BF_10_ = 22.39, with a larger congruence effect in Austria (76.84 ± 66.41 ms) than in China (32.42 ± 49.44 ms). This difference was significant as shown by a Welch’s *t*-test, with *t*(79.4) = 3.54, *p* = 0.001, *d*_between_ = 0.76, 95% CI [0.32, 1.19], BF_10_ = 42.51. The results for the Macanese group lie in-between Austria and China, with a congruence effect of 63.23 ± 54.66 ms, which, with a Bonferroni-corrected level of *α* = 0.05/3 = 0.017, did neither differ significantly from Austria (*t*[52.9] = 0.90, *p* = 0.374, *d*_between_ = 0.22, 95% CI [– 0.29, 0.72], BF_01_ = 2.83) nor China (*t*[41.3] = 2.25, *p* = 0.030, *d*_between_ = 0.60, 95% CI [0.08, 1.12], BF_10_ = 2.43).

**Fig. 2:**
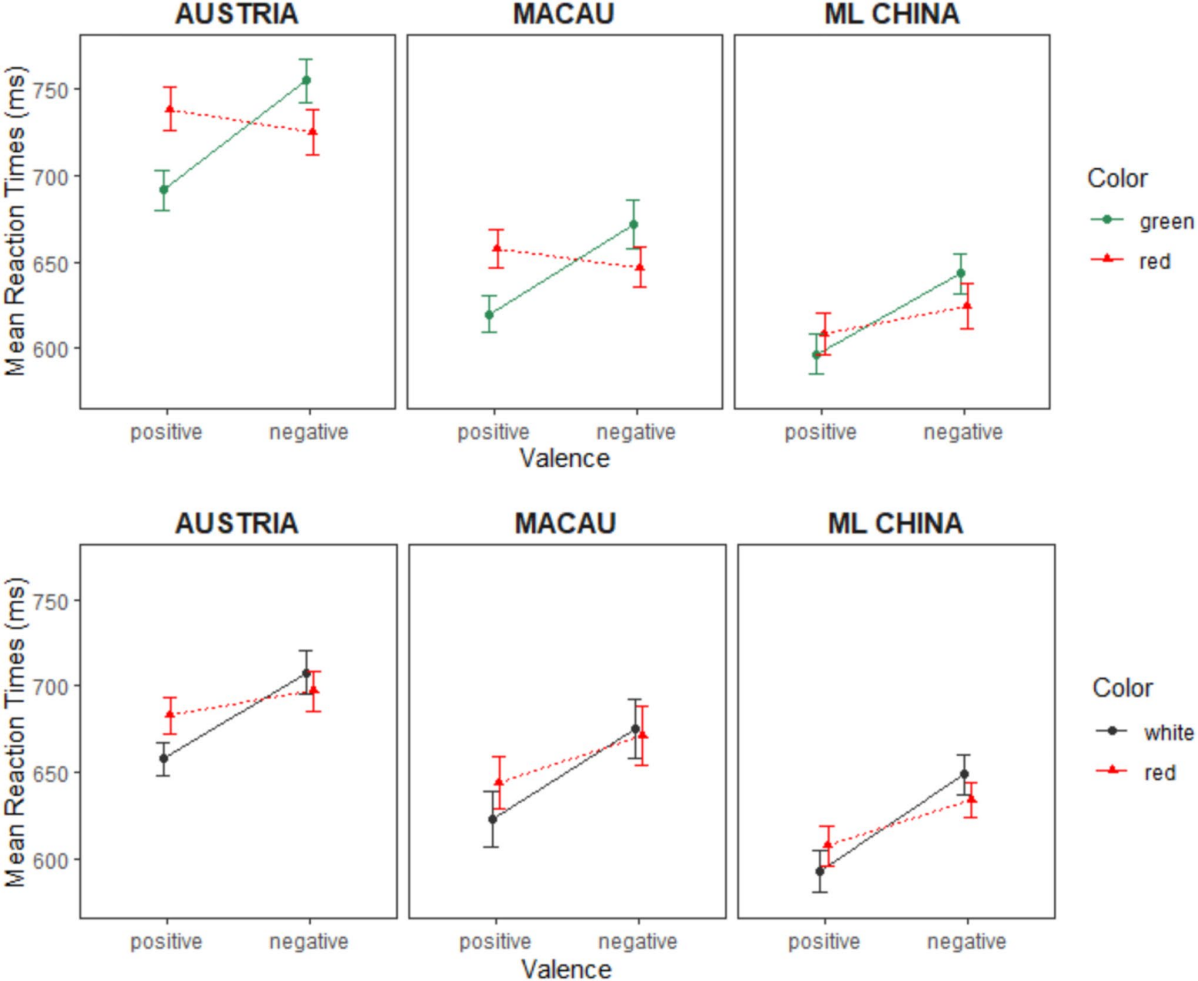
Means of the median correct reaction times in Experiment 1 for positive and negative words per Country Group. The upper panel shows response times in the red–green color opposition block. The lower panel shows response times in the red–white color opposition block. Error bars indicate SEMs

The means are plotted in Fig. 2 (upper panel) and the three-way interaction is visualized as the distance between the data points (means of the median correct RTs). Note that we are interested in the overall congruence effect, to which each factor combination of color and valence contributes (incongruent or red-positive minus congruent or red-negative; incongruent or green-negative minus congruent or green-positive, see Section “Predictions and the congruence effect measure”).

##### Red–white color opposition

We analyzed RT data from the 106 participants who started the experiment with the red–white color opposition block (47 from Austria, 42 from China, 17 from Macau)^7^ and ran a repeated measures ANOVA, with Country as a three-level between-participants factor (Austria vs. China vs. Macau), and Color (red vs. white) and Valence (positive vs. negative) as within-participant factors.

We found a significant main effect for Country in the red–white block, *F*(2, 103) = 10.76, *p* < 0.001, $\eta_{{\text{p}}}^{2}$ = 0.173, 90% CI [0.067, 0.270], $\eta_{{\text{G}}}^{2}$ = 0.155, BF_10_ = 492.36, with faster responses from China (611.57 ± 70.58 ms) and Macau (640.73 ± 49.54 ms) than Austria (684.64 ± 70.91 ms). The main effect for Valence was also significant again, *F*(1, 103) = 87.61, *p* < 0.001, $\eta_{{\text{p}}}^{2}$= 0.46, 90% CI [0.341, 0.549], $\eta_{{\text{G}}}^{2}$ = 0.06, BF_10_ = 2.85 × 10^24^, with faster categorization times for positive (633.13 ± 77.13 ms) than negative words (664.62 ± 78.13 ms). Color showed a significant main effect, *F*(1, 103) = 4.19, *p* = 0.043, $\eta_{{\text{p}}}^{2}$= 0.039, 90% CI [0.001, 0.116], $\eta_{{\text{G}}}^{2}$= 0.001, BF_01_ = 3.24, with responses to red being slightly slower than to white stimuli (655.57 ± 77.22 ms vs. 648.54 ± 76.47 ms, respectively). The Color × Valence interaction was significant as well, *F*(1, 103) = 48.50, *p* < 0.001, $\eta_{{\text{p}}}^{2}$ = 0.32, 90% CI [0.200, 0.423], $\eta_{{\text{G}}}^{2}$= 0.012, BF_10_ = 9.74 × 10^4^. However, in this case, the interaction was *not* modulated by Country, *F*(2, 103) = 0.40, *p* = 0.669, $\eta_{{\text{p}}}^{2}$ = 0.008, 90% CI [0, 0.042], ${\upeta }_{{\text{G}}}^{2}$< 0.001, BF_01_ = 8.59. Thus, the CEs were similar between countries, with 36.05 ± 53.67 ms in the Austrian group, 29.18 ± 39.77 ms in the Chinese, and 25.69 ± 42.16 ms in the Macanese group (all Welch’s *t*-tests showed *p* > 0.43). For all other effects *F*s < 2, all *p*s > 0.20. Means of the median correct RTs for all country groups are illustrated in Fig. 2 (lower panel).

Table 1 contrasts CE sizes per country across both color systems (red–green, red–white) for Experiments 1 and 2. Nominally, for group Austria and Macau, CEs in the red–white block were smaller than in the red–green block, while for China, the CEs were similar in size across both color systems. For an illustration of the respective CE sizes per color block and country, see Fig. 4 (left panel). Generally, the analysis of the error rates corroborated the RT findings (see online supplementary material).

**Table 1:** Comparison of mean congruence effects (in ms) per Color System and Country over Experiments 1 and *2*

	China		Austria/Germany		Macau	
	Red–Green	Red–White	Red– Green	Red–White	Red–Green	Red–White
Exp. 1—Words	32.42	29.18	76.84	36.05	63.23	25.69
Exp. 2—Silhouettes	25.97	25.97	43.08	37.83	–	–

### Discussion

Results from Experiment 1 provided key evidence for a cultural contribution to color-valence associations: Only in a red–green color system did association strength diverge (with a stronger implicit green-positivity/red-negativity association for Western than for Chinese participants). No cross-cultural differences in association strength were found in a red–white color system—a condition in which findings from explicit association studies would have predicted larger dissimilarities (see Section “Predictions and the congruence effect measure”). The results from Experiment 1 will be discussed in more detail in the General Discussion (Section “General discussion”).

One potential confound in the results of Experiment 1 and in particular in the found cultural differences relates to the stimuli being words. This is a complication, as words are not strictly the same in Chinese and German. In fact, our data show that Chinese speakers—Mandarin and Cantonese alike—categorized word valence faster than speakers of German did. Importantly, general differences of word processing between languages might play a role in our observed group differences. Both Asian groups use the Chinese character writing system. Several studies suggest an advantage of the time it takes to access semantic information of a word in Chinese over languages using the Latin alphabet, with evidence from reading times (Lü & Zhang, 1999) and semantic preview benefits in eye-tracking studies. This advantage is due to the fact that “the Chinese writing system is based on a closer association between graphic form and meaning than is alphabetic script” (Yan et al., 2009, p. 561).^8^ Note that the mean length of all used German words was 6.83 letters or 2.18 syllables. The mean length of the Mandarin words was 2.07 characters or 18.06 strokes. Of course, the Country main effect might also reflect a general, stimulus-independent processing advantage of Chinese over Austrian participants. We suspected, however, that it is more likely a linguistic artefact. Importantly, if meaning (i.e., semantic information about the affective valence of a word/concept) is extracted very quickly when reading Chinese characters, the color information carried *with* the linguistic cue might not be as effective in facilitating (in case of congruent color-valence pairings) or inhibiting (in case of incongruent pairings) lexical access, semantic retrieval and response execution. Interestingly, when looking at the results of the Macanese group, in particular in the red–green context, we found a greater similarity to color-valence association patterns of the Austrian group, but less overlap with the Mainland Chinese results. One might argue that this already speaks against a merely word-processing based interpretation of the found cultural differences. However, to confirm differences in implicit cross-modal associations between cultures *independently* of word-processing differences between these cultures, we used color-manipulated pictorial images instead of words in Experiment 2.

## Experiment 2

Experiment 2 was carried out as an online study and tested native Mandarin-speakers (from China) and native German-speakers (from Austria and Germany) in two color opposition blocks: red–green and red–white. Generally, we expected to observe similar congruence effects to Experiment 1—that is, we did expect cultural differences in the red-green color system, but not in the red–white color system. In addition, if cultural differences in color-valence associations in Experiment 1 were due to word-processing differences between the Chinese and the German language, no culture-dependent differences in color-valence congruence effects were to be expected in Experiment 2, with its pictorial stimuli.

### Methods

In Experiment 1, the data from Macau was useful in confirming a general main effect for Chinese (Mandarin/Cantonese) speakers over German speakers. However, to determine the influence of language on the previous results, one Chinese sample was sufficient for Experiment 2.

#### Participants

Data from a total of 251 participants was collected online. For the Chinese-speaking group, 124 participants were recruited through an advertisement that was posted via WeChat to the open group of the psychology laboratory of Shaanxi Normal University, Xi’an, China. Chinese participants were rewarded with 20 CNY for valid participation.^9^ For the German-speaking group, 46 participants from the University of Vienna were recruited in return for (partial) course credit.^10^ An additional 81 participants (students with Austrian or German nationality) were recruited via Prolific (www.prolific.co) and paid 2.20 GBP for valid participation, resulting in a total of 127 German-speakers. Participants were randomly assigned to one of the conditions that resulted from permuting block order and key location.

The same exclusion criteria applied as in Experiment 1. From the collected data (*n*_GER_ = 127, *n*_CH_ = 124), we excluded participants with an accuracy rate lower than 75% (*n*_GER_ = 0, *n*_CH_ = 2), a failed color discrimination test (*n*_GER_ = 9, *n*_CH_ = 9), or a reported birth place other than Germany or Austria (*n*_GER_ = 7) for the German speakers or Mainland China (*n*_CH_ = 1) for the Mandarin speakers. This left data from a total of 223 participants, 112 subjects (age = 19.8 ± 4.4; 30 male) in the Chinese group and 111 subjects (age = 24.1 ± 4.3; 57 male) in the German group. Note that, contrary to Experiment 1, we will refer to the latter sub-sample as "German group" and use the label "Germany" in the plots of Experiment 2 for simplicity's sake. The sample consisted of 25 Austrian and 86 German nationals.

#### Materials

*Silhouette selection* Eighty positive and 80 negative silhouettes (300 × 300 px) were taken from the Bicolor Affective Silhouettes & Shapes (BASS) database (https://gasparl.github.io/BASS; Kawai et al., 2021). The BASS is well suited for comparing a Western culture and China, since it contains representative valence and arousal ratings from both cultural groups, with the Western (in the BASS database, US) ratings presumed to be comparable with Austrian ratings.^11^ We carefully controlled for culturally comparable valence and arousal ratings from the West/US and East/China. This means specifically, that the Western/US valence and Chinese valence ratings were similar on average among the positive silhouettes as well as among the negative silhouettes. At the same time, there was enough within-category heterogeneity between different silhouettes both in the positive and negative categories, both on the side of the Western/US ratings and the Chinese ratings. Regarding arousal levels from the Western/US sample, they were similar for positive and negative silhouettes, but this was impossible to accomplish for Chinese arousal ratings, due to the stronger linear valence-arousal-relationship among Chinese participants (with positive silhouettes being rated as more arousing than negative ones). However, we reduced the difference as much as possible. Lastly, the number of black and white pixels was comparable between positive and negative silhouettes, which means that the amount of color present (red, green, or white, depending on condition, see next paragraph) was similar across both valence conditions. A compilation of these mean values is available in Table 3 of the Appendix. The full list of the 160 silhouettes we used is available in the online supplementary material.

*Color manipulation* Similar to the procedure of Experiment 1 (for Chinese/Macanese participants), Experiment 2 consisted of two consecutive blocks with color-manipulated stimuli: a red–green block and a red–white block, only now, instead of linguistic material, the stimuli were pictorial. Black pixels of the original black-on-white silhouettes were replaced with red, green, and white color. Shades of red and green were taken from Wilms and Oberfeld (2018), with similarly high saturation/lightness: red (L*a*b* [50, 81.29, 82.05] = RGB [245.63, 0, 0]) and green (L*a*b* [50.03, − 98.59, 55.35] = RGB [0, 148.83, 0]). White pixels (i.e., the background) of the original silhouettes were replaced with a mid-gray (L*a*b* [50, 0, 0] = RGB [119, 119, 119]). Note that, since the experiment was run online, we were not able to control the monitor settings of the participants’ setup. However, at the beginning of the experiment, we asked participants to set their monitor brightness to the highest level, and we included a short color discrimination test as well (see Procedure) to make sure that the colors we manipulated were discernible.

#### Procedure

The experiment had to be completed in a Google Chrome Browser.

*Color vision test* Before the experimental task, we showed three pictures of the Ishihara number plates (500 × 500 px) as a first screen-out test. In addition, four rectangles (200 × 100 px) colored in the shades of the red and green we used for the silhouettes, as well as one brown tone (“Saddle brown”, RGB [139, 69, 19]) and one olive-green tone (“Olive Drab”, RGB [107, 142, 35]), were presented and participants were asked to select the color they saw for each rectangle. Only if participants entered all three numbers from the Ishihara plates and the colors of the rectangles correctly, they could proceed with the experiment.

*Valence categorization task* Participants were then presented with the informed consent and, except for the study on Prolific, were asked to provide demographic data (age, gender, place of origin). Thereafter, they saw the instructions (in German for German speakers, in Simplified Chinese for Mandarin speakers) that informed them about the upcoming task (i.e., that they will see a series of silhouette pictures in different colorations, and need to press key “E” for positive images, and key “I” for negative images, or vice versa). To make the valence category clear to the participants, all 80 positive and 80 negative silhouettes were shown (in black-on-white) before the task started. After successful completion of a practice round, the two experimental blocks, the red–green and red–white block, were presented (in counterbalanced order). Per block, each silhouette was shown twice—once in each color. This resulted in a total of 80 (positive) + 80 (negative) = 160 * 2 (in Color 1 + in Color 2) = 360 * 2 (Block 1 + Block 2) = 720 experimental trials.

In both experimental blocks, silhouettes were presented in the center of the screen against a darker gray background (RGB [112, 112, 112]). When no answer was logged within 2 s after stimulus onset, the message “Too slow!” (in the participant’s language) was shown for 500 ms and the stimulus disappeared. If the participant gave an incorrect response within the response window, the message “Incorrect!” (in the participant’s language) was shown for 500 ms and the stimulus stayed on screen until the correct response was given. After the correct response was logged, the next trial started (i.e., the next silhouette was displayed). Between the two experimental blocks, participants could take a break of self-determined length and were informed of the altered color of the upcoming stimuli.

#### Data analysis

One silhouette (*falling.png*) was excluded from analysis due to being classified incorrectly over 40% of the time in the German-speaking subject group (and over 35% in the Mandarin-speaking subject group). The exclusion of this stimulus did not affect the analysis results to a meaningful extent. For all analyses, only the first response to each stimulus presented was used, and all practice trials were excluded. From all valid 223 participations, responses below 150 ms and above 2 s were discarded (990 out of 142,720 trials, i.e., 0.69%). For RT analysis, only correct responses were used (discarding an additional 11,122 of all remaining 141,730 trials, i.e., 7.85%).

### Results

Data from both culture groups was collected in two conditions that differed in the presentation order of the experimental blocks (red–green block first vs. red–white block first). Since analyses showed that Block Order was not a determining factor in the three-way interactions (Color × Valence × Country) we were most interested in (see supplementary analysis in online material), we disregarded block order in the analyses presented below.

With the mean correct response times (RTs), we ran repeated measures ANOVAs for each color block, with Country (West vs. China) as between-participants factor, and Valence (positive vs. negative) and Color (red vs. 2nd color) as within-participant factors. Again, we first report the results from the analyses of the red–green color block and then from the red–white color block. RT means and *SD*s can be found in Table 1. The analyses of the error rates are available in the online supplementary material.

#### Red–green color opposition

We found significant main effects for the factors Valence and Color, with *F*(1, 221) = 110.66, *p* < 0.001, $\eta_{{\text{p}}}^{2}$ = 0.334, 90% CI [0.252, 0.406], $\eta_{{\text{G}}}^{2}$ = 0.023, BF_10_ = 3.72 × 10^30^, and *F*(1, 221) = 64.68, *p* < 0.001, $\eta_{{\text{p}}}^{2}$ = 0.226, 90% CI [0.150, 0.301], $\eta_{{\text{G}}}^{2}$ = 0.006, BF_10_ = 1.64 × 10^7^, respectively, showing faster responses to negative (719.02 ± 87.58 ms) than to positive silhouettes (748.23 ± 100.32 ms)—a valence effect going in the other direction compared to word stimuli—and faster responses to red (726.42 ± 93.93 ms) than to green silhouettes (740.83 ± 91.72 ms). Interestingly, the main effect for Country was not significant in the silhouette categorization (but it was significant with words in Experiment 1), *F*(1, 221) = 0.27, *p* = 0.605, $\eta_{{\text{p}}}^{2}$ = 0.001, 90% CI [0, 0.020], $\eta_{{\text{G}}}^{2}$ = 0.001, BF_01_ = 2.29.

**Fig. 3:**
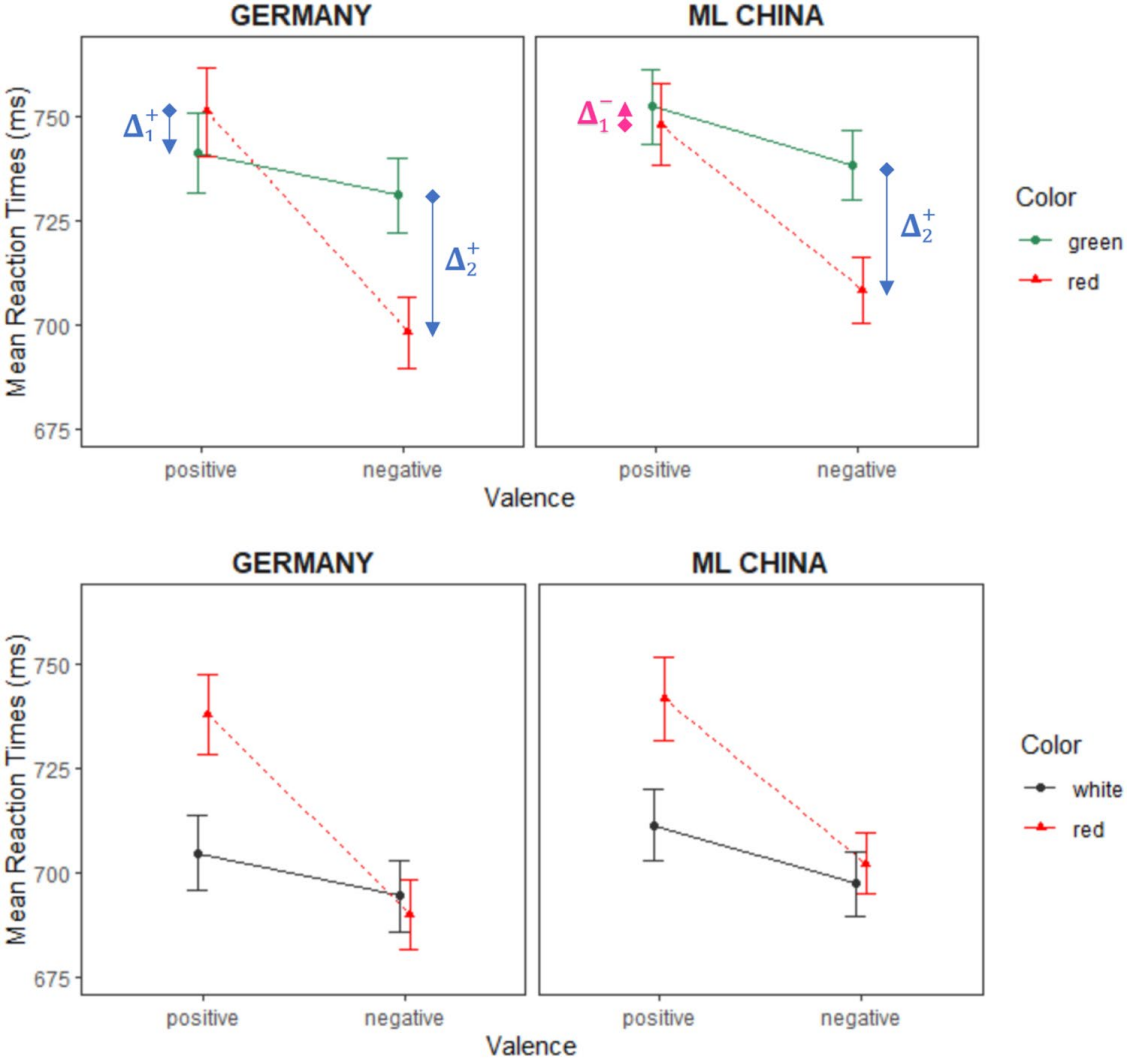
Mean reaction times for positive and negative silhouettes in Experiment 2 per Country Group (Germany versus Mainland China). The upper panel shows response times in the red–green color opposition block. The lower panel shows response times in the red–white color opposition block. Error bars indicate SEMs. To illustrate the CE in the Red-Green Condition (upper panel), blue arrows are drawn from Incongruent (black diamond) to Congruent (black inverted triangle) Color-Valence pairings, showing the category differences Δ_1_ and Δ_2_. Per Country group, the overall CE = Δ_1_ + Δ_2_, the sum of the category differences. If responses to Incongruent (black diamond) stimuli are slower than to Congruent (black inverted triangle) stimuli, then the difference Δ is positive ( +). If the inverse is true, the Δ is negative (−), reducing the summed/overall CE (depicted as a pink arrow). Note that the arrow is inverted in the Chinese Red-Green positive condition, making the Incongruent pairing (marginally) faster than the Congruent pair, leading to one inverse (= negative) term Δ_1_, reducing the overall Chinese CE

The Color × Valence interaction was significant, *F*(1, 221) = 94.08, *p* < 0.001, $\eta_{{\text{p}}}^{2}$ = 0.299, 90% CI [0.218, 0.372], $\eta_{{\text{G}}}^{2}$ = 0.008, BF_10_ = 6.24 × 10^11^. Most importantly, this interaction was modulated by Country, resulting in a significant three-way interaction, with *F*(1, 221) = 5.79, *p* = 0.017, $\eta_{{\text{p}}}^{2}$ = 0.026, 90% CI [0.002, 0.069], $\eta_{{\text{G}}}^{2}$ < 0.001, BF_10_ = 1.04. For the German group, *M* ± *SD* of the mean CE size was 43.08 ± 54.84 ms, for the Chinese group 25.97 ± 51.31 ms (raw mean difference: 17.11 ms, 95% CI [0.43, 27.52]). Welch’s *t*-test showed that the distributions between the two groups differed significantly, *t*(219.7) = 2.40, *p* = 0.017, *d*_between_ = 0.32, 95% CI [0.06, 0.59], BF_10_ = 2.18. All other interactions were not significant (all *F*s < 2.60, *p*s > 0.10). Figure 3 (upper panel) illustrates the RT data.

#### Red–white color opposition

**Fig. 4:**
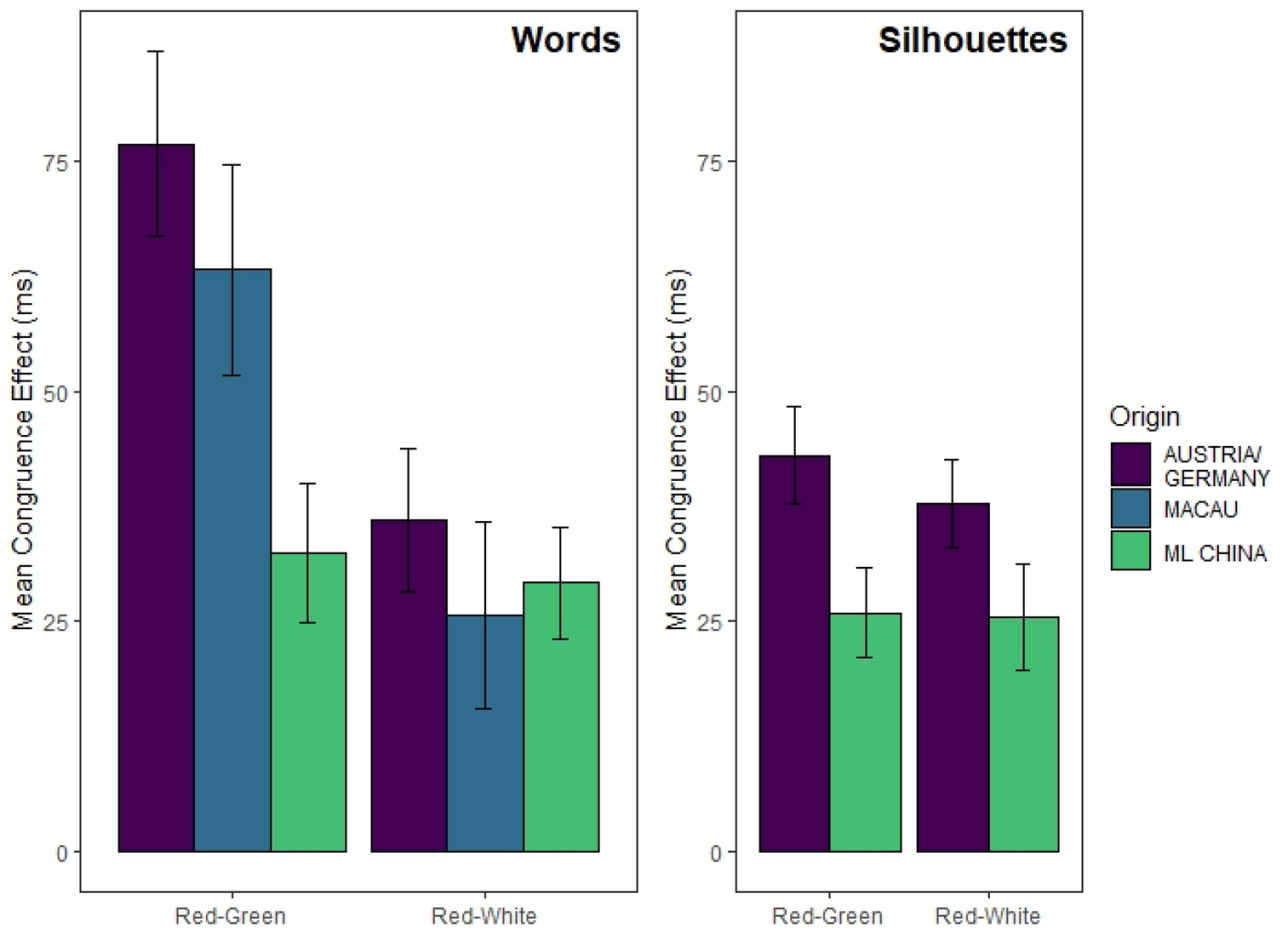
Reaction-time based mean congruence effects (Incongruent Minus Congruent Performance in ms) in Experiment 1 (Left Panel) and Experiment 2 (Right Panel) per Color System and Country. Error bars indicate SEMs

Main effects for Valence and Color were significant, with *F*(1, 221) = 93.53, *p* < 0.001, $\eta_{{\text{p}}}^{2}$ = 0.297, 90% CI [0.217, 0.371], $\eta_{{\text{G}}}^{2}$ = 0.023, BF_10_ = 6.22 × 10^26^, and *F*(1, 221) = 75.31, *p* < 0.001, $\eta_{{\text{p}}}^{2}$ = 0.254, 90% CI [0.176, 0.329], $\eta_{{\text{G}}}^{2}$ = 0.008, BF_10_ = 4.41 × 10^8^, respectively. While the Valence effect was in the same direction as previously, in this color block, responses to red (717.97 ± 89.15 ms) were slower than to white silhouettes (701.90 ± 86.26 ms). Just as in the red–green block RTs, the main effect for Country was not significant, *F*(1, 221) = 0.31, *p* = 0.579, $\eta_{{\text{p}}}^{2}$ = 0.001, 90% CI [0, 0.021], $\eta_{{\text{G}}}^{2}$ = 0.001, BF_01_ = 2.61. The interaction between Color and Valence was significant, *F*(1, 221) = 71.88, *p* < 0.001, $\eta_{{\text{p}}}^{2}$ = 0.245, 90% CI [0.167, 0.320], $\eta_{{\text{G}}}^{2}$ = 0.007, BF_10_ = 1.07 × 10^9^, but—as opposed to the RTs in the red–green block—not significantly modulated by Country, *F*(1, 221) = 2.74, *p* = 0.099, $\eta_{{\text{p}}}^{2}$ = 0.012, 90% CI [0, 0.047], $\eta_{{\text{G}}}^{2}$ < 0.001, BF_01_ = 2.17. CE sizes in the red–white block were 37.83 ± 50.55 ms for the German and 25.97 ± 51.31 ms for the Chinese group. Welch’s *t*-test showed no evidence for a significant difference, *t*(221.0) = 1.74, *p* = 0.083, *d*_between_ = 0.23, 95% CI [–0.03, 0.50], BF_01_ = 1.66. For an illustration of the respective mean RT congruence effect sizes per color block and country in Experiment 2, see Fig. 4 (right panel). All other interactions were non-significant as well (all *F*s < 0.75, *p*s > 0.25). The mean RTs for the red–white block are illustrated in Fig. 3 (lower panel). The analysis of the error rates confirmed the effects from the red–white color system, but the three-way interaction in the red–green color system was not significant (for more details see online supplementary material).

### Discussion

Experiment 2 showed that implicit color-valence associations also show with non-linguistic, pictorial stimulus material. In general, the data supports a cultural contribution to implicit associations and again, color systems were found to play a significant role: In the red–green color system, congruence effects were weaker among Chinese than among German participants, while they did not differ (i.e., were equally ‘weak’) between cultures in the red–white color system. However, the observed association strength for word stimuli (Experiment 1, especially for the red–green color system in the Western group) was substantially stronger than in Experiment 2, reflecting to the overall smaller cross-cultural differences for silhouette stimuli (we compare and discuss the results of the two experiments in more detail in the following General Discussion).

## General discussion

In the current study, we investigated the possible origin of color-valence associations as either culturally specific or universal. We compared samples from Eastern (China) and Western cultures (Austria and Germany), as well as a culture group which unites Eastern and Western cultural influences, namely Macau (in Experiment 1).

In general, we found that, whether the stimulus is lexical or pictorial, our participants exhibited a great overlap in implicit color-valence associations across cultures. This was particularly true when color-valence associations were tested in red–white color opposition blocks (as compared to red–green color opposition blocks). Here, regardless of culture, our participants showed a trend for faster categorization of white-positive and red-negative pairings. An important cross-cultural difference that our implicit measures identified could be observed in the red–green color system. We showed that the red-negative/green-positive association was significantly stronger in the Western than in Chinese groups. Responses from the Macanese participants lay in-between these groups, but generally overlapped more with the Western than with the Chinese sample. Both of these findings—cultural similarity in a red–white system and cultural difference in a red–green system—stand in contrast to anything we would have expected on the basis of existing research with explicit measures (i.e., homogenous explicit emotion associations for the colors red and green across cultures, but stronger explicit sadness associations for the color white by Chinese participants, see Jonauskaite et al., 2020a).

### Polar opposites and polarity correspondence

Our findings highlight the significance of color systems. Their influence was reflected in the fact that culture dependencies were present for one and the same color, red, only if it was opposed to green, but not if it was opposed to white.^12^ Accordingly, the presence of green highlights a more negative meaning of red, just in our Western and not in the Chinese culture samples. Interestingly, this negative semantic shift in red associations does not happen to an equal extent with an opposing white in the color system. Response times in Experiment 1 demonstrated that in a red–white color system, associations between red and positivity come out more prominently, even in the Austrian sample.^13^

A possible explanation is that the red–green opposition fostered the dominance of the red-negativity association among Western participants only, potentially by some type of idiosyncratic connection, such as the German sayings with positive meanings incorporating the color green (e.g., “Grün ist die [Farbe der] Hoffnung”, *green is the [color of] hope*; or “Alles im grünen Bereich”, *everything is alright, lit. ‘in the green range’*) and, in turn, boosting the negative valence of the color red as its contextual opposition. Our results confirm that green is a unanimously positively associated color across cultures, but it seems that its presence can tip the scale for the ambiguous red towards predominantly negative associations in the West. Red might simply be perceived as more of a threat cue in the West because it is repeatedly used as a danger signal, most often when green is present and takes on the opposite (positive) meaning. This specific finding would be in line with the color-in-context theory in general (Elliot & Maier, 2012) and with the influence of color systems in particular. A recent study by Nadarevic et al. (2021) brought forward additional evidence for the role of color context, showing in a series of color-Stroop tasks that, for German-speakers, a red-false association only emerges in a context of opposed green-true, but not gray-true.

The dimension-specificity hypothesis by Schietecat et al., (2018a, 2018b) predicts the results from the Western group well: A larger CE reflected a larger conceptual distance on the valence dimension of the color pair red and green than of the pair red and white. But how can the culture-dependence of the red–green (but not the red–white) color system be understood? The dimension-specificity hypothesis does not give us any more clues as to how to understand the particular origin in cultural experiences of this specific cross-cultural difference. Explanations for why we did not observe a similar shift in red-associations depending on an opposing green in China might be that the color opposition is not used to the same extent in terms of message signaling. A predominance of positive associations in Chinese culture and possibly a greater exposure to red in non-threatening contexts in general (e.g., red lanterns, street lights and building illumination; red coloring for hits in search results in internet search engines, etc.) might have stabilized red-associations and prevented evoking equally strong negative (e.g., threat) cue perception in the Chinese compared to the Western participants. In the Introduction (Section “Predictions and the congruence effect measure”) we also hypothesized that a red–white color system could function as a prominent alternative in Chinese message signaling, which may cause red to be less of an ‘antipole’ to green (relatedly see the polarity correspondence principle, e.g., Proctor & Cho, 2006). However, we did not find any evidence that would speak for a stronger (‘more prototypical’) red–white than red–green opposition in the Chinese group (especially not with white being more negatively associated than red, see above).

What gives rise to the mappings of colors onto semantic dimensions in the first place? If we follow grounded cognition accounts, then human knowledge is based on sensory information collected from our environment (e.g., Barsalou, 2008; Havas & Matheson, 2013; Williams et al., 2009). In the case of color-valence associations, we would naturally look at how color is used—physically and in language—and base our culture comparison on differences and similarities we find there. Thus, in theory, explicit association studies might reveal a good deal of ideas about multisensory anchoring processes and potential points of divergence between cultures. However, explicit measures are limited by what can be expressed (awareness) and what happens to be expressed (willingness, response restriction/selection); and judging by our findings of an absence of a culture-specific ‘emotional white effect’ as well as a presence of a culture-specific red–green-opposition effect, some underlying information might not come to light through explicit measures alone. Practically, one would, therefore, have to look beyond explicit measures, as the implications of culture-specific ‘color-in-context’ findings for applications are important in themselves: Color opposition regarding valence (and other semantic dimensions) can conceivably play a role in utility research and everyday tasks. As an example, think of the usage of color systems with green and red buttons on a control board for go versus stop responses, respectively. By showing clear differences to explicit measures—more cross-cultural similarities for the color white, less cross-cultural similarities for the color red—the current implicit measure study reinforces the view that explicit and implicit approaches can yield complementary results and that both should be taken into account when planning applications. Without more systematic research on cross-cultural differences and similarities in implicit measures of color-valence and color-emotion associations, one can otherwise not easily predict which of several possible associations dominates in a particular context (cf. Elliot & Maier, 2012). The present results, thus, support the value of implicit measures as an additional source of information besides the usage of explicit measures for understanding color-valence associations in general and how they work in color opposition systems in particular.

### Words versus pictures

Chinese speaking participants did respond faster to words than did German speaking participants. The absence of a Country main effect in Experiment 2 suggests that the speed advantage for Chinese speakers found in Experiment 1 was likely related to processing linguistic stimuli (Chinese characters versus Latin alphabet). At the same time, the fact that Experiment 2 replicated the selectively stronger CE for West over China rules out that the cross-cultural asymmetry in implicit associations was (solely) based on processing idiosyncrasies related to the Chinese language/writing system.

Despite the converging general findings of selectively stronger CEs for the West in red–green color systems, there were also some peculiar results in Experiment 2 that are worth pointing out. The first concerns the ‘negativity bias’: In Experiment 1, negative words elicited *slower* responses and *more* errors, whereas in Experiment 2, negative silhouettes elicited *faster* responses and *fewer* errors. This finding is not without precedents. Other studies have shown that negative content captures attention efficiently (e.g., ‘Automatic Vigilance Effect’, Pratto & John, 1991) by drawing cognitive resources away from the analysis process and subsequent response execution when conveyed in word form (under similar paradigms as ours, e.g., Ansorge & Bohner, 2013; Meier et al., 2004; Moller et al., 2009), but capturing attention and allocating resources towards faster response selection when conveyed in pictorial form (De Houwer & Hermans, 1994; Mogg et al., 2000; Schimmack, 2005; but see Giner-Sorolla et al., 1999; Ihssen & Keil, 2013). The second finding concerns the ‘ambiguity of red’: In Experiment 1, the difference between RTs to positive-red and negative-red words was notably smaller compared to words in green or white, which led us to conclude that red is a largely ambiguous color (see also the previous studies reviewed in Section 1). However, for red silhouettes, RT differences between positive and negative stimuli were considerably more pronounced, suggesting a generally strong (universal) red-negativity association when it comes to pictorial material. It is possible that silhouettes and contours of natural objects provide physical cues to object color (cf. Hansen et al., 2006; Tanaka & Presnell, 1999)—an influence obviously lacking with words. T. Wang et al. (2014) conducted a study investigating *naturally* versus *culturally* motivated red-valence associations, under the rationale that verbal color terms (in contrast to physical color cues) are more reflective of, and possibly more sensible to, cultural idiosyncrasies, due to being more abstract in nature, and are less reflective of associations based on experiences in natural environments. We used physical color throughout all experiments, but the notion still applies when comparing our results from words with that of the more natural silhouettes depicting real objects (cf. Wang et al., 2014). This explanation would also account in part for the generally smaller CEs in the silhouette study that would arise due to the more universal associations occurring in experience with natural objects than with the more disparate culture-dependent social experiences with and associations of words with valence and/or colors. Note that, contrary to Experiment 1, negative–positive RT differences in green and white silhouettes are rather small in general, suggesting that participants did not universally hold negative connotations for these colors. This is also in accordance with previous research (Lakens et al., 2012; Moller et al., 2009).

### Limitations

The online experiment (Experiment 2) poses the problem of less reliable RT measurement, but, fortunately, reliability was at least high enough for a replication of the general pattern found under more controlled laboratory conditions (i.e., in Experiment 1). In addition, as mentioned earlier, we did not have strict control over the apparatus and actual colorimetrics throughout both studies (usage of different laboratories in different countries in Experiment 1, online study in Experiment 2). The slower reactions (and higher error rates, see online supplementary material) for green silhouettes (color main effect) could reflect problems with the presentation of this color. This might be an artefact of the online setting, despite our efforts to equate red and green in lightness and saturation and testing participants’ color discrimination ability. In any case, it would be desirable to run a similar study in the future under more rigidly controlled laboratory conditions, to ensure consistent color presentation and viewing conditions. Such a study could also help to tell the influences of the different color dimensions to the currently measured color-valence congruence effects apart (cf. Schloss et al., 2020). The reported experiments were a collaborative group project and all authors had the chance to contribute to the interpretation of the results and the implications of the study. However, the first author wishes to disclose her positionality as white researcher with Western background and acknowledges the possibility for unintended biases at every stage of the research process.

For this study, we tested participants from Austria and Germany—both countries which might be subsumed culturally under the umbrella term "Western". Whether our results and conclusions generalize to other Western countries, remains to be tested. Similarly, China is a region rich in linguistic and cultural variety. The results of the research reported here should be contextualized accordingly.

## Conclusion

Color-valence associations are important for many applications. The current study showed that color-valence associations can be assessed through various modes of delivery—linguistic and pictorial. However, not all color-valence associations apply universally and in each context of color systems, so caution is advised when using them in international contexts and together with alternative colored signals. For Western populations, association strength of negative-red and positive-green is stronger than for Chinese participants. The red–green opposition seems, hence, particularly effective (in terms of polarity attributions) in the West. In comparison, a red–white opposition seems to allow for relatively weaker red-negativity associations from a Western viewpoint, but would work equally efficient from a Chinese viewpoint.

## Data Availability

All studies were preregistered using the OSF (Foster & Deardorff, 2017), accessible via https://osf.io/27gf8/. The supplementary analyses, source code for the experiments, R analysis scripts, materials used, and all data collected are available via the same link. For all experiments, all measures, conditions, data exclusions, and data acquisition practices were reported.

## Appendix

See Tables 2 and 3

**Table 2:** Means and SDs of the Mean Response Times (in ms) for Experiments 1 and 2

		Red–Green color block				Red–White color block			
		Red		Green		Red		White	
Experiment	Country	Negative	Positive	Negative	Positive	Negative	Positive	Negative	Positive
1 – Words	China	628 ± 86	613 ± 83	645 ± 77	598 ± 76	631 ± 66	605 ± 77	646 ± 75	590 ± 79
	Macau	647 ± 56	658 ± 53	672 ± 67	620 ± 50	671 ± 69	644 ± 62	675 ± 71	623 ± 66
	Austria	725 ± 88	738 ± 83	755 ± 83	691 ± 79	697 ± 78	683 ± 72	708 ± 85	658 ± 69
2 – Silhouettes	China	708 ± 84	748 ± 102	738 ± 90	752 ± 95	702 ± 78	742 ± 106	697 ± 83	711 ± 90
	Germany	698 ± 89	751 ± 111	731 ± 94	741 ± 101	690 ± 86	738 ± 101	694 ± 90	705 ± 95

In Experiment 1, we show the means of the median correct RTs, and only for the first experimental block performed by the participant.

**Table 3:** Mean Valence and Arousal Rating Scores (US and China) and the Average of Colored (i.e., Not Background-Colored) Pixels for the Positive and Negative Silhouettes used in Experiment 2

Valence	Count	Arousal US	Arousal China	Valence US	Valence China	Colored pixels (out of 90,000)
Negative	80	5.260	4.671	3.408	3.759	21,906
Positive	80	5.036	5.653	6.949	6.531	22,176
Average		5.148	5.162	5.178	5.145	22,041

## Footnotes

1 Here, we diverge somewhat from our preregistration which states that we will follow up a significant three-way interaction (Color × Valence × Country) with Color × Valence repeated measures ANOVAs for each between-subjects group (i.e., Country), since we believe that congruence effects are a more comprehensive measure of comparison.

2 When color words are used, it is unclear which exact color participants imagine, as each color label covers a range of physically different colors (cf. Lillo et al., 2018). In addition, with visual color words instead of real colors, there is also a complicating influence of incongruence between word color and word-color meaning. For example, in Jonauskaite et al., (2020b), all color words were shown in the same achromatic presentation color (gray/black), meaning that they were cast in incongruent colors relative to their meaning at times. In comparison to color words, the current experiment, thus, at least allowed some control over color appearances (cf. Wang et al., 2014).

3 Throughout the paper, we use “Macanese” as an adjective denoting “from Macau” or “pertaining to Macau”. We do not use the adjective in reference to the ethnic group (Macanese People). We also do not use it as a proper noun that denotes the Creole language.

4 If the number of words per category was lower than 50, the shortest word list (list of words rated positive or list of words rated negative by the participant) determined the overall number of stimuli that appeared in the subsequent task. For an equal number of positive and negative items, the word list of the remaining valence category was trimmed accordingly. For example, if a participant rated 43 of the 60 positive words (per BAWL-R database) as positive and 52 of the 60 negative words as negative, then the 43 positive-rated words were selected for the participant’s valence-categorization task, as well as the same number of negative-rated words, in which case the total number of items would be 43 + 43 = 86 items. Note that the number of positively rated (e.g., 43) and negatively rated words (e.g., 52) does not necessarily have to add up to 120. In the example, 43 out of 60 positive words were “correctly” judged by the participant as, in fact, positive, while 17 of the 60 positive words were judged as negative. Furthermore, the participant judged 52 out of the 60 negative words as, in fact, negative, but judged eight of the negative words as positive (e.g., ‘naïve’ is a negative term according to the BAWL-R database, but could be judged as a more positive concept by some participants).

5 Note that this is a non-standardized assessment of the Ishihara test for color deficiency and may not give accurate results as for the presence or absence of color-blindness. However, since our experimental setup also made use of computer-display colors, it was important to us to verify if participants were able to discriminate shades of red and green on a computer monitor. So we opted to include this control measure, in addition to self-reported color deficiencies.

6 Originally, the analyses with a three-leveled factor Country were preregistered as supplementary. The main analysis was originally planned as focusing only on an Austria versus China comparison. Since we report the three-country comparison here, we report the two-country comparison as secondary analysis in the supplementary online material.

7 In the preregistration, we said that we wanted to test at least 43 participants per country. However, due to exclusions, the number of participants in China was actually only 42, and Macau only 17. As mentioned above, the analyses with Country as a two-level factor (Austria vs. China) showed very similar effects (available in the online supplementary material).

8 Chinese words are composed of one or more characters (logograms); and each character, in turn, is composed of one or more constituents that each carry meaning. This results in the sublexical units of the Chinese writing system having “more direct contact with meanings” (Perfetti et al., 2002, p. 36).

9 The amount of monetary reimbursement we preregistered per participant was lower (10 CNY). We decided to increase the amount to give sufficient incentive for Chinese participants because they saw the advertisement on their mobile device but were not allowed to run the study on it. Instead, they had to use a PC to participate in the study.

10 We preregistered to open 120 places for online participation for students from the University of Vienna only. After a span of one month, no more students volunteered to take part in the study, so we completed participant collection via Prolific. The results from Austrian students and from German-speaking Prolific participants are highly similar and, in fact, give the same key results in comparison with the Chinese results even when tested separately.

11 Taking the mean ratings from the Austrian BASS-norming pilot study (*n* = 78), the correlation between the Austrian and the US mean valence ratings for the 583 pictures was very high with *r*(581) = .0.928, 95% CI [0.915, 0.938]. The correlation between the groups’ mean arousal ratings was somewhat lower than for valence but still high, with *r*(581) = .0.807, 95% CI [0.777, 0.834].

12 Looking only at the red trials from Experiment 1, the difference between the (red) negative and the (red) positive response latencies was significantly different between Austria and China *only* in the red-green opposition condition (*M* ± *SD* in ms = –1 3.40 ± 48.96 vs. 14.86 ± 40.63, respectively; *t*(82.8) = – 2.93, *p* = .0.004, *d* = – 0.63, 95% CI [–1.06, – 0.19], BF_10_ = 8.62), but not in the red–white opposition condition (*M* ± *SD* in ms = 14.14 ± 49.56 vs. 26.56 ± 41.69, respectively; *t*(86.7) = – 1.28, *p* = .0.203, *d* = – 0.27, 95% CI [– 0.69, 0.15], BF_01_ = 2.22).

13 In the Austrian sample, the RT differences between positive red and positive green trials in the red-green opposition block (*M* ± *SD* = 46.99 ± 44.43) were significantly larger than the differences between positive red and positive white trials in the red–white opposition block (*M* ± *SD* = 25.15 ± 34.60), raw mean difference: – 21.84, 95% CI [– 38.52, – 5.15]); *t*(81.2) = – 2.60, *p* = .0.011, *d* = – 0.55, 95% CI [– 0.97, – 0.13], BF_10_ = 4.30.
